# A Sensor Concept for Direction-Selective Monitoring of Partial Discharges in Medium-Voltage Switchgears

**DOI:** 10.3390/s26092672

**Published:** 2026-04-25

**Authors:** Bastian Zimmer, Frank Jenau, David Ripka, Nils Porath

**Affiliations:** Department of Electrical Engineering and Information Technology, Institute of High Voltage Engineering, TU Dortmund University, 44227 Dortmund, Germany; frank.jenau@tu-dortmund.de (F.J.); david.ripka@tu-dortmund.de (D.R.); nils.porath@tu-dortmund.de (N.P.)

**Keywords:** partial discharge (PD), condition monitoring, high-frequency current transformer (HFCT), magnetic flux concentrator sensor (MFCS), direction selective, experimental setup

## Abstract

**Highlights:**

**What are the main findings?**

- The introduced sensor concept, Magnetic Flux Concentrator Sensor (MFCS), is used for partial discharge (PD) monitoring and is less susceptible to saturation effects than a typical High-Frequency Current Transformer (HFCT).
- A method for evaluating measured partial discharges to estimate the direction of the fault location is presented. This method needs two sensors, the MFCS and an HFCT.

**What are the implications of the main findings?**

- With the MFCS, the stable monitoring of PD, independent of the load current, is possible, making it very reliable for trend analysis.
- With the direction-selective method, PD is monitored with more precision in medium-voltage networks.

**Abstract:**

Knowledge about the condition of electrical equipment in energy networks is of great importance to network operators. Partial discharges are a key parameter for evaluating the health of the insulation. While a quantifiable PD measurement for offline tests is state of the art, it is costly and labour-intensive. It, therefore, makes sense to carry out permanent monitoring during operation. At the medium-voltage level in the European interconnected grid, comprehensive monitoring of PD is not implemented. This study presents a novel sensor concept that is used to detect PD in medium-voltage switchgear and cables: the so-called Magnetic Flux Concentrator Sensor (MFCS). It is an inductive sensor concept with high sensitivity in the frequency range of a few MHz, like well-established High-Frequency Current Transformers (HFCTs) but with better magnetic saturation properties in specific use cases. The highly permeable ferrite core of the MFCS is unconventionally shaped, resulting in a higher-saturation field strength. Therefore, this sensor is not driven into saturation by the operating currents of typical MV power cables. Using the MFCS and conventional HFCT in a suitable combination enables direction-selective PD detection. This work presents the sensor concept and the method for directional detection of the PD location, as analysed and evaluated theoretically and practically with laboratory experiments.

## 1. Introduction

Due to global warming, over 196 countries agreed to reduce the global warming effect to a maximum of 2 °C in the Paris Agreement [1]. As a result, more renewable energy generation is incorporated into the European interconnected grid. In Germany, this is most impactful at the medium-voltage level (typically 10 kV to 30 kV) due to two reasons. Firstly, most PV panels are installed at the LV to MV levels, and, secondly, the connection between wind turbines is typically realized with medium-voltage cables [2]. Therefore, monitoring of medium-voltage cables becomes more important.

An established method for condition monitoring is the detection and analysis of partial discharges. PDs are local discharges that do not bridge the whole insulation but only a part of it [3,4]. PDs occur when the electrical field strength is higher than the strength of the electrical insulation of the medium; for example, this can happen when there are air cavities in a solid insulation medium. PDs are typically a side effect of damaged or aged high-voltage equipment [3]. While there are standardised methods for measuring PD, such as the standard IEC 60270, these methods are costly and labour-intensive, making them unsuitable for monitoring purposes and better suited for laboratory or on-site offline tests [4]. For online-condition monitoring, PD detection is possible with different sensors. In particular, HFCTs are commonly used for condition monitoring of power cables [5,6,7]. A typical HFCT is a special type of current transformer with one primary winding and several secondary windings. The high-frequency currents resulting from PDs are electrically insulated and magnetically coupled by a ferrite core, typically nickel–zinc or manganese–zinc. With the standardised method, the apparent charge of the PD is determined, while unconventional methods, such as detection with HFCT, rely on the analysis of the output voltage, often without further calculation of the charge. This study focuses on the evaluation of voltage as the direct output of the PD sensor, particularly since the sensor is used for PD trend detection, meaning determination of the absolute PD level is not necessary.

**Figure 1:**
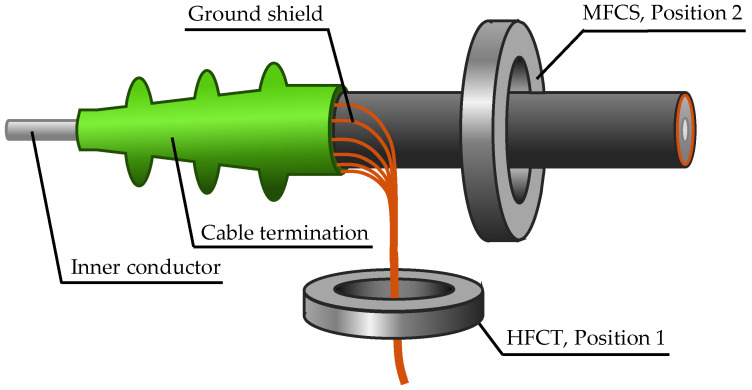
Possible inductive sensor core positions of the HFCT and MFCS for PD detection in medium-voltage cables.

There exist two basic sensor mounting positions for cable systems. The most common is around the grounded cable shield [8], and the second, less common position is around the entire cable [6]. Figure 1 illustrates the two positions.

HFCTs with ring cores and thin air gaps are typically used in position 1. Air gaps of less than 1 mm are a common occurrence and have been extensively documented in the literature [6]. Position 2 is characterised by the disadvantage that the core of the transformer is driven into magnetic saturation by the high magnetic field of the rated current. The implementation of air gaps has been demonstrated to be an effective solution to this issue by reducing the magnetic flux. In a recent study from 2023, air gap dimensions that result in an operation without saturation are characterised for these mounting positions [9]. Specifically, air gap lengths of up to 3 mm are analysed, and an HFCT that is suitable for rated currents up to 300 A was developed [10,11]. Another common option is the use of Rogowski coils, i.e., inductive sensors in the form of air coils without a highly permeable core [12,13]. A Rogowski coil is characterised by high linearity and cannot be saturated but has very low output voltage levels, especially for PD detection compared to HFCTs. Consequently, the utilisation of amplifier circuits is typically necessary [13,14].

This study describes and analyses an alternative sensor approach. The introduced MFCS is based on the use of an unconventional core geometry in the form of a half-ring core. This so-called flux concentrator was first introduced by the authors in [15,16] and is a promising alternative with good saturation behaviour and less material usage, making it more cost efficient. This study focuses on a comparison between typical HFCTs and the novel MFCS under realistic conditions. As the main difference between the MFCS and an HFCT is the core geometry, this study analyses the influence of the modified geometry. To ensure the greatest possible comparability, HFCTs with different air gaps and the MFCS are constructed using the same core materials and the same windings. Consequently, it is solely the geometric parameters which are varied. For a detailed characterization of the MFCS in comparison to the HFCT, a realistic laboratory setup is constructed so that the PD measurement sensitivity is evaluated under real operating conditions. Additionally, the saturation characteristics are analysed and compared for the MFCS and the HFCT.

Furthermore, the innovative use case for the MFCS is that **both sensor positions** are used **in combination** to enable **direction-selective PD detection**. The method introduced in this study is based on the evaluation of the polarity of the measured PD impulses, thus diverging considerably from the established methods explained in the following sections.

In the context of PD localization, time-based methods are most relevant [17]. These methods utilise the finite propagation speed of electromagnetic traveling waves as a result of PD. The established methods are limited to time domain reflectometry (TDR), the phase difference method, time of arrival (ToA) and the analysis of the time parameters of individual pulses [17]. The TDR method is characterised by high robustness with short cables; however, its application is constrained to offline situations, as the cable must be disconnected from the network [18,19,20]. The phase difference method is similar to TDR and is based on the analysis of the phase shift between the direct and reflected signals in the frequency domain. Studies have demonstrated that this method is reliable for offline use, but its efficiency is reduced in offline use [21].

Methods that can be used online include time of arrival (ToA) and analysis of the time parameters of the pulses. With ToA, PD sensors are used at both ends of the cable to measure the resulting pulses for a specific PD event with a time delay, depending on the distance of the fault location from the respective sensor. The location of the fault is determined by this time difference. This method is suitable for online use because it is not based on reflection events and, therefore, does not need to be as sensitive as TDR. The disadvantage is the significant reliance on sensor time synchronisation [22].

By analysing the time parameters of individual PD pulses, referred to in the literature as the rise time or transfer function method, the rise time of a PD pulse is evaluated to draw conclusions about the distance of the fault location. The underlying physical principle is that an impulse undergoes greater distortion with increasing propagation time due to effects such as dispersion and attenuation. However, this requires sensors with a very large bandwidth, ideally in the GHz range [23,24].

These methods focus on estimating the location of PD inside a cable. There is no method to distinguish between PD originating from inside the cable versus the switchgear that is based on the polarity of PD impulses. In this study, an approach is presented to estimate the direction of the fault location based on the evaluation of the polarity. This method requires a sensitive sensor to be placed around the entire MV cable. Two main research questions are raised and answered in this study:

**1.**  **How is an inductive high-sensitivity PD measurement without saturation possible and what are the characteristics of such a sensor?**
**2.**  **How can the direction of the PD source location be estimated using this sensor concept?**

**Figure 2:**
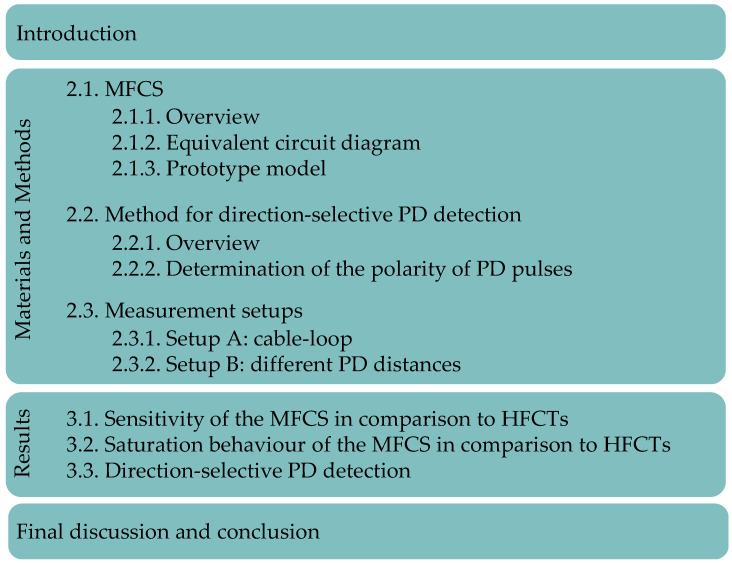
Structure of this study.

To answer these questions, an analytical and experimental approach is chosen. In the following sections, the MFCS is described and analysed in a laboratory setup to quantify its sensitivity and the saturation characteristics. Furthermore, the direction-selective PD detection method is analysed experimentally using this sensor. An overview of the structure of this study is given in Figure 2.

## 2. Materials and Methods

In the following subsections, the used materials, especially the sensor configurations, are described. Afterwards, the method of direction-selective PD detection is presented. Lastly, the measurement setups in the high-voltage laboratory used for validation are explained.

### 2.1. MFCS

In this subsection, the relevant topics regarding the MFCS are presented, starting with an overview, continuing with qualitative modelling, and finalised with the introduction of prototype models.

#### 2.1.1. Overview

The name Magnetic Flux Concentrator Sensor comes from the so-called flux concentrator, which is a special type of transformer core. This term is not commonly used in the context of inductive measuring transducers and sensors and will be explained in more detail below. HFCTs, typically used in PD sensor technology, are constructed with highly permeable ferrite cores, in which thin air gaps in the sub-millimetre to low-millimetre range are commonly used, as described in the Introduction. In this study, the term HFCT refers to all high-frequency transformers with thin or no air gaps.

To distinguish clearly between a typical HFCT and an MFCS, the term *MFCS* is used to describe an inductive high-frequency transformer based on a flux concentrator as its core. In this case, the flux concentrator is a half-ring core. Electromagnetically, no closed, highly permeable magnetic circuit is created. Instead, the magnetic stray flux is reduced in comparison to air core coils; more precisely, the magnetic flux is concentrated through the secondary winding. This is where the name flux concentrator derives from.

#### 2.1.2. Equivalent Circuit Diagram

**Figure 3:**
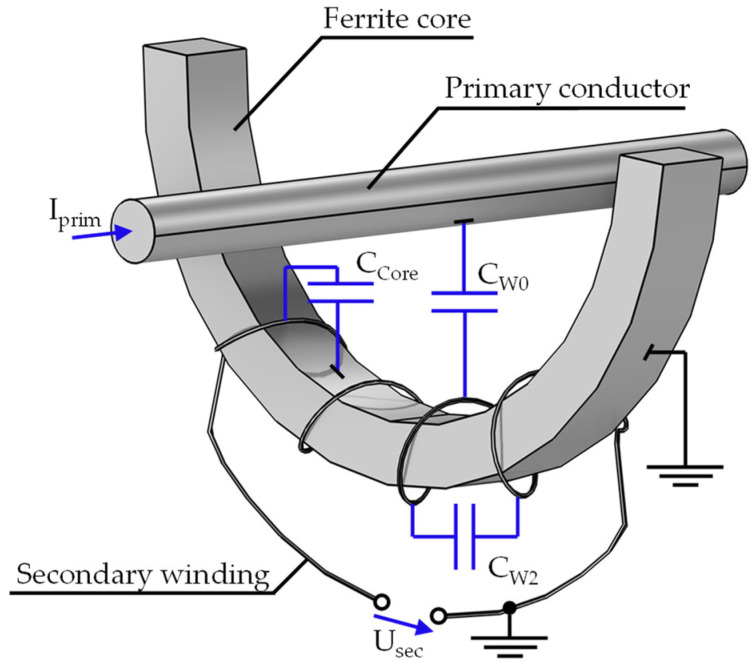
Visualization of the MFCS with a half-ring core. The parasitic capacities are coloured blue.

In the following, an equivalent circuit diagram is derived to discuss further properties qualitatively. This diagram is similar for MFCS and HFCT. Both variants have a highly permeable core made of a ferromagnetic material, around which the secondary winding is wound. In most applications, the primary winding is formed by the cable to be measured, meaning the primary turn count is 1. On the secondary side, few turns are used, as increasing the number of turns causes unwanted parasitic effects that reduce sensitivity. It is advantageous to keep the winding capacity low. Since an MFCS is operated at high frequencies, typically in the range of 100 kHz to 10 MHz, the parasitic capacitive couplings between the winding, core and ground have an increasingly significant influence on measurement accuracy with increasing frequency. In order to illustrate the parasitic capacities, the qualitative representation from Figure 3 is utilised.

The capacitance C_W0_ is the total capacitance between the primary and secondary windings. C_W2_ comprises the capacitance between the individual turns of the secondary winding. The capacitance C_Core_ forms between the secondary winding and the grounded ferrite core.

**Figure 4:**
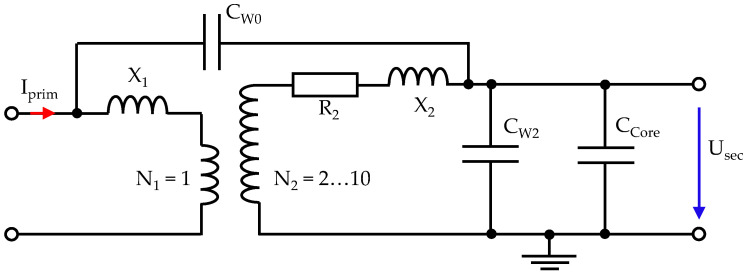
Equivalent circuit diagram for the MFCS with parasitic capacities.

The equivalent circuit diagram is based on typical transformer diagrams, as presented in [3], but the ohmic primary conductor losses are neglected because the primary turn number is 1. Additionally, the parasitic capacities are added, resulting in an adjusted diagram for the MFCS in Figure 4.

This diagram clearly shows that, at high frequencies, the capacities C_W2_ and C_Core_ become low impedance and behave like a quasi-short circuit on the secondary side. This ultimately reduces the amplitude of the secondary voltage and, thus, the sensitivity of the sensor. Therefore, the secondary winding needs to be optimised for the given purpose, with the aim of achieving the highest possible inductance while keeping the capacities as low as possible [5,6]. The details regarding the turn numbers are explained in the next subsection.

#### 2.1.3. Prototype Model

The following section describes the specific design of a laboratory prototype model for the inductive sensors. Both the final laboratory prototypes for the MFCS and for the various HFCT configurations are based on the same prototype. This ensures maximum comparability when analysing different air gap dimensions.

Highly permeable cores are available on the market as toroidal cores in a variety of shapes and sizes. For practical reasons, a toroidal core is preferred. The dimensions are an inner radius of 80 mm, an outer radius of 96 mm, and a thickness of 10 mm. These half-ring cores are used to create closed-ring cores, as well as a half-ring cores for the MFCS and various air gaps for the HFCT. The air gaps are created using PMMA (polymethyl methacrylate) films, which are layered to different thicknesses. With a relative permeability of approximately 1, PMMA behaves magnetically like air [25].

Ferrites are typically used as the core material for HFCTs due to their high-frequency properties. In this study, manganese–zinc (Mn-Zn) is used as the core material. It has high permeability and high electrical volume resistance, which dampens eddy currents.

As discussed in the previous section, the secondary winding involves a compromise between high inductance and low winding capacity. This is generally confirmed by reviews from recent years [5,6]. Specifically, some publications conclude that fewer turns lead to higher sensitivities for PD detection, with the optimal number of turns being less than 20 [6,13,26,27,28,29]. A more differentiated analysis concludes that a greater number of turns shifts the sensitive frequency range of the sensor towards lower frequencies [26]. However, the exact number of turns required depends on the desired sensor bandwidth, which is highly dependent on the core material. In this study, the experimentally determined optimal number of turns is 4.

To sum up, the final laboratory sample consists of one ring half for the MFCS and two half rings for the HFCT, which are held together by a housing. PMMA is used as the air gap between the halves. Both sensor concepts are created with 4 turns for the secondary winding, and Mn-Zn ferrite is used as the core material. The final air gaps are shown in Table 1.

### 2.2. Method for Direction-Selective PD Detection

#### 2.2.1. Overview

**Figure 5:**
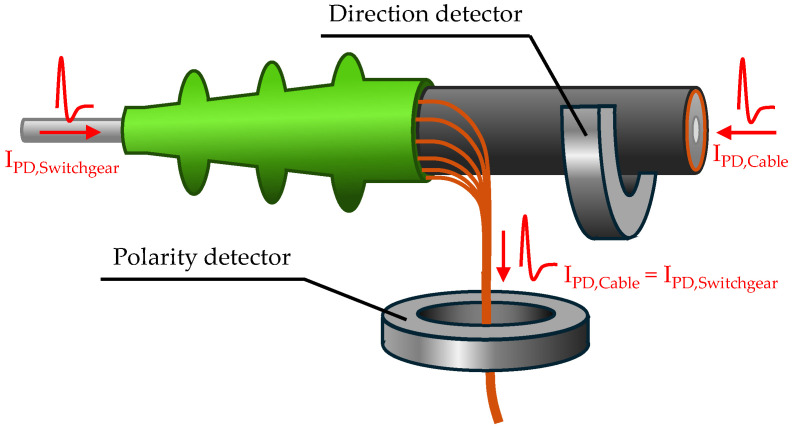
Visualisation of the sensor positions for the polarity and direction detection.

The novel method presented for direction-selective PD detection is based on the combined use of two PD sensors. One sensor acts as a polarity detector and the second sensor as a direction detector. The standard configuration is visualised in Figure 5.

To explain further details, the direction detector will be considered first. It is designed as an MFCS that is installed around the whole MV cable, as shown in Figure 5. Assuming an idealised current pulse is generated by a partial discharge in the cable system or in the switchgear, this current will flow through the effective area of the MFCS. Via magnetic coupling, a secondary voltage is induced at the output terminals of the MFCS, according to Formula (1), which corresponds to the negative of the time derivative of the current pulse.

(1) $$u_{ind}=-N\cdot L_{K}\frac{di_{TE}(t)}{dt}$$

This means that the first half-wave of the induced voltage always has the opposite polarity to the original current pulse. Therefore, the polarity of the induced voltage can be used to determine the polarity of the current pulse.

However, this does not yet provide any information about the direction. In order to achieve this objective, it is necessary to undertake a more detailed examination of the manner in which PD pulses propagate. As a result of partial discharges, PD pulses propagate from the PD source in the form of electromagnetic traveling waves in the system. The current traveling waves and the voltage traveling waves are always linked [3]. According to [3], the solution to the equations is that the voltage traveling wave is described with:

(2) $$u\left(z,t\right)=U\cdot f\left(z-vt\right)+U\cdot g\left(z+vt\right)$$

And a current traveling wave is described with:

(3) $$i\left(z,t\right)=I\cdot f\left(z-vt\right)+\left(-I\right)\cdot g\left(z+vt\right)$$

The waves propagate in both the positive and negative *z*-direction, described with the term *f*(*z* − *vt*) for the positive *z*-direction and the term *g*(*z* + *vt*) for the negative *z*-direction. Here, *v* is the propagation velocity of the wave and *t* is time.

At a constant voltage, it is noticeable that the associated current waves have different signs in different directions, meaning that the current direction is reversed. Considering that the polarity of the induced voltage at the inductive sensor is always opposite to the polarity of the current pulse, positive current traveling waves from the positive *z*-direction and equally positive current traveling waves from the negative *z*-direction induce opposite voltages at the inductive sensor. Provided that the installation direction and winding of the sensor are known, it is possible, with additional knowledge of the polarity of the current pulse, to deduce the origin direction of the pulse from the polarity of the induced voltage.

At this point, the role of the second sensor, the polarity detector, becomes apparent. It is assumed that a pulse of any polarity from any direction always flows in the same direction to the earth contact. Experimental investigations confirm this and show that the polarity of the induced voltage of a sensor in the earth path depends solely on the polarity of the pulse itself. This means that current sensors in the earth path of the system are suitable for detecting polarity.

Assuming PDs occur either in the switchgear or in the cable, the induced voltages at both sensors will differ depending on the polarity, as shown in Table 2.

To sum up, with the same polarity of the induced voltage at both detectors, the origin of the PD must always be at the opposite location to that with different polarities. This is independent of the PD polarity. Consequently, **a polarity comparison of both detectors is sufficient to determine the direction of origin of the PD in relation to the direction detector**. The prerequisite is knowing the installation direction and winding of the detectors. The polarity comparison happens in two steps:

First, determine the polarity of the impulse with the polarity detector (HFCT).

Second, determine the direction of the impulse with the direction detector (MFCT).

#### 2.2.2. Determination of the Polarity of PD Pulses

The polarity of the pulses, some of which oscillate strongly, can be determined from the polarity of the first half-wave. Analysing the first half-wave has the advantage that distortion of the measured pulse due to the superposition of reflected waves does not occur. In the case of heavily oscillating pulses, especially when the PD origin is further away, it is not always possible to clearly determine the polarity using a threshold value. Therefore, this study considers an analytic approach using the Hilbert transform for polarity detection.

The Hilbert transform is used in communication systems. According to [30,31], the Hilbert transform of a signal s(t) is defined as the convolution of s(t) and $\frac{1}{\pi t}$ as follows.

(4) $$s_{H}\left(t\right)=\frac{1}{\pi t}*s(t)$$

With the Hilbert transform, the analytical signal $s_{A}(t)$ is calculated with Equation (5).

(5) $$s_{A}\left(t\right)=s\left(t\right)+js_{H}(t)$$

The analytic signal is of a complex form, where the imaginary part is the Hilbert transform and the real part is the original signal. In audio signal processing, the magnitude $\left|s_{A}(t)\right|$ of the analytical signal is often used, since it is the complex envelope of the original signal, as in Formula (6) [32,33,34].

(6) $$\left|s_{A}(t)\right|=\sqrt{{s(t)}^{2}+{s_{H}(t)}^{2}}$$

**Figure 6:**
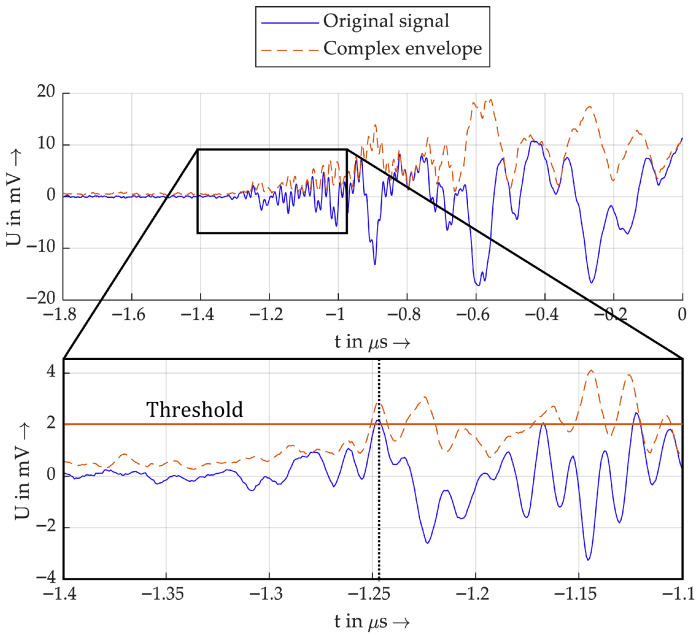
Example of the peak detection with the Hilbert transform.

This is especially used to detect the polarity of audio signals in [32]. For polarity detection, the peaks of the envelope $\left|s_{A}(t)\right|$ are detected, and afterwards, the deviation of the Hilbert transform is evaluated at the time of the peak of the envelope. This is presented as a robust method for positive or negative peak detection, since $\left|s_{A}(t)\right|$ is often not as noisy as the original signal. In the context of this study, the polarity detection of the first half-wave of the measured impulses is performed similarly. At first, a threshold value is defined. Then, the first local maximum of the complex envelope $\left|s_{A}(t)\right|$ that exceeds the threshold value is detected. To determine the polarity, the polarity of the original signal at the time of the peak value of the envelope is evaluated. An example of this is shown in Figure 6.

The blue original signal oscillates strongly and has a positive and a negative peak at approximately −1.25 µs and −1.225 µs. Therefore, it would be highly dependent on the threshold value whether this negative or positive peak value would be detected as the first half-wave. The complex envelope shows a peak value at the time of both peak values, with similar amplitude. In this example, the first is detected, and the raw signal is positive at this point, indicated here by the black dotted line.

**Figure 7:**
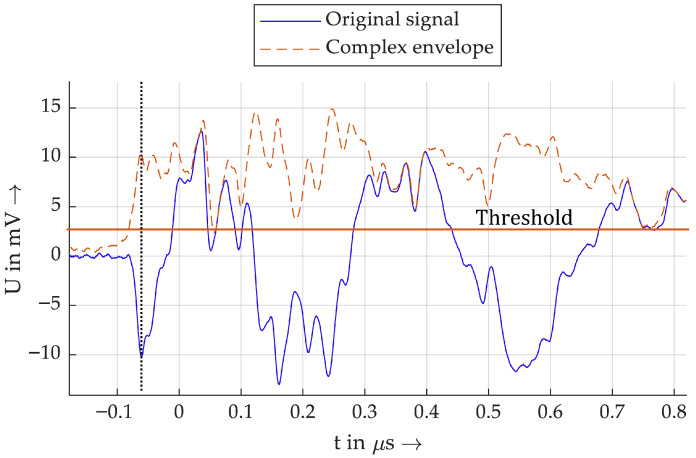
Example 2 of a PD impulse.

Figure 7 shows another example a PD impulse. Although the polarity is clearly recognizable here based on the original signal, this also works with the envelope method.

For the validation, both algorithms are implemented, one based on the Hilbert transform and the complex envelope and the other based on the original signal. The first one is called Hilbert algorithm and the second is called standard algorithm.

### 2.3. Measurement Setups

For the scope of this study, two measurement setups are used. One consists of a closed MV cable loop to enable the induction of high rated currents (setup A). The other setup consists of a single MV cable line to test with PD at different distances (setup B).

#### 2.3.1. Setup A: Cable Loop

This section is devoted to a laboratory setup that enables the analysis of PD sensors, particularly the MFCS, in comparison to the HFCT, under nominal conditions. The setup is characterised by the following key features:

- Real medium-voltage cable with a fault location for PD generation.
- Real positioning of the PD sensors on the medium-voltage cable.
- Generation and measurement of an AC voltage of 12 kV.
- Simultaneous generation and measurement of an AC load current at the maximum current-carrying capacity of the MV cable.

**Figure 8:**
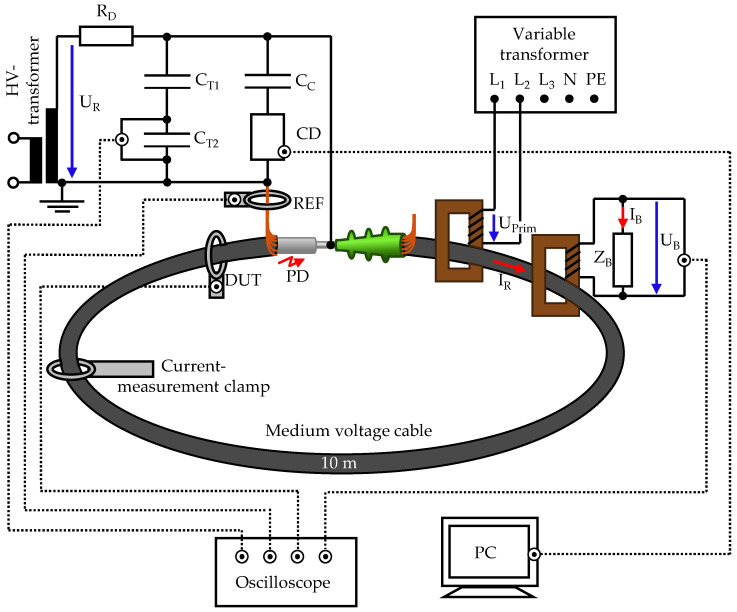
Overview of setup A with the integrated cable loop.

To meet these requirements, an MV cable loop is integrated into a high-voltage setup. The load current is fed inductively into the cable. The entire setup is shown in an overview diagram in Figure 8.

The medium-voltage cable is a 12/20 kV cable with VPE insulation and an aluminium inner conductor of type NA2XS(F)2Y.

The inner conductor of the cable is short-circuited. The shield is grounded on one side and connected to the ground of the rest of the high-voltage system. The other side of the cable shield is not grounded so that no closed circuit is created via the ground.

A heating transformer is used to induce a 50 Hz load current *I_R_* in the cable loop. This has several windings on the primary side to which a voltage is applied. The secondary winding is formed by the medium-voltage cable. On the primary side, a permissible input voltage of 230 V ± 10% and a maximum current consumption of 8.7 A are specified. On the secondary side, this results in a maximum effective current of 1000 A. The heating transformer is supplied via a three-phase variable transformer. This allows for adjusting the phase angle of the load current in relation to the high voltage.

The current is measured in two ways. Firstly, the effective value of the load current is measured using a current clamp, which has a measurement accuracy of 5%. Secondly, the instantaneous value of the current is determined using a current transformer. This is a transformer identical in design to the heating transformers but operated in reverse to function as a measuring transformer. On the secondary side, a measuring load of 228.5 mΩ is installed. The rated current *I_R_* is calculated using the transformation ratio of the transformer r = 115, the load resistance, and the measured load voltage *U_B_*.

The high voltage *U_R_* is generated by a single-phase high-voltage transformer. The primary side of the transformer is fed via a variable transformer. A damping resistor *R_D_* = 50 kΩ is installed directly behind the test transformer to limit the short-circuit current in the event of a fault.

The applied high voltage is measured using a capacitive voltage divider with an upper capacitance of *C_T_*_1_ = 100 pF and a lower capacitance of *C_T_*_2_ = 1.8 µF.

Only one end of the MV cable is fitted with a cable termination. The other end is open, serving as a PD source. Partial discharge measurement in accordance with IEC 60270 takes place in parallel. A coupling capacitor *C_C_* is used for this purpose, through which the high-frequency PD signals are coupled out of the test object.

The inductive PD sensors HFCT and MFCS are mounted at the open cable end and around the grounded conductor. The positions are marked with DUT and REF. Since PDs have stochastically fluctuating amplitudes and are not guaranteed to remain constant over several measurement series, a reference HFCT is used at the REF position for all measurements. For each PD pulse, the amplitude of the reference HFCT is compared with that of the HFCT or MFCS to be analysed at the DUT position. Overall, this makes it possible to record individual PD pulses simultaneously with the reference and the DUT. Further details will follow in the subsequent sections of the respective measurement series.

#### 2.3.2. Setup B: Different PD Distances

The studies conducted to validate and analyse the direction-selective PD measurement method require a laboratory setup with PD in different locations. This setup is similar to the one described in Section 2.3.1, except that no load current is generated, and the MV cable is not constructed as a closed loop. Only one end of the cable is connected to the HV laboratory setup.

**Figure 9:**
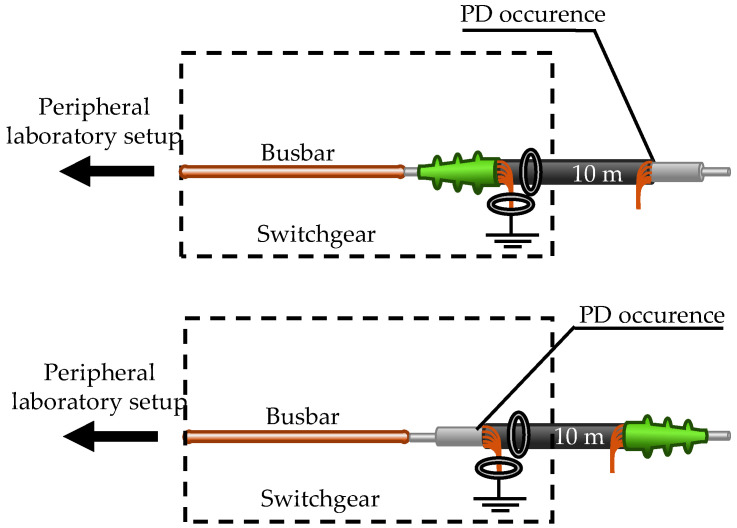
Two possible configurations with the short cable to generate PD directly at the inductive sensors or at a distance of 10 m.

The HFCT and MFCS are always positioned at the start of the cable. A 10 m cable section is used, which is terminated at one end and open at the other end. There are two possible configurations, as shown in Figure 9.

**Figure 10:**
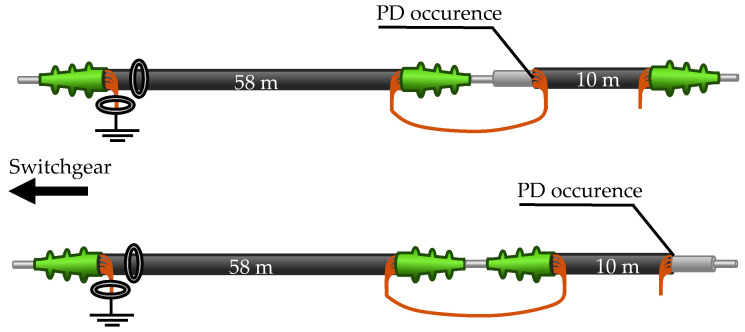
Configurations with both cables for PD at 58 m or 68 m distance.

Furthermore, there is a 58 m cable section that is free of PD. This makes it possible to generate PD at a cable distance of 58 m or 68 m. Figure 10 shows the possible cable combinations. It should be noted that in all cable configurations, the cables are always only grounded on one side. The shield at the distant end of the cables is not grounded separately.

## 3. Results

In this section, the results of the main measurements are presented. This is divided into three subsections that focus on the sensitivity of the MFCS, the saturation characteristics of the MFCS, and the analysis of the direction-selective PD detection.

### 3.1. Sensitivity of the MFCS in Comparison to HFCTs

This section compares the sensitivity of the MFCS with that of several HFCT configurations featuring different air gap dimensions. The aim is to analyse the transfer behaviour under defined and reproducible operating conditions.

During the measurements, the total current through the sensor consists of the rated current *I_R_* and the partial discharge current *I_PD_*. When the load current is switched on, the magnetic operating point of the sensor shifts on the hysteresis curve. This effect is directly related to the saturation characteristics and is relevant for evaluating sensitivity. The aim of this section is to evaluate the sensitivity of the laboratory samples listed in Table 1. The measurement procedure is divided into the following three steps:

- Switching on the load current.
- Switching on the high voltage.
- Setting the test voltage (maximum 12 kV).

The test voltage is selected to ensure that the partial discharge pattern (PRPD) remains consistent across the various measurement series. The PD pulses are recorded at a sampling rate of 1.25 GHz. A time window of 8 µs is recorded for each pulse. Triggering is performed via the reference sensor (REF), as this does not saturate and, thus, ensures reliable pulse detection. A total of 150 individual PD pulses are recorded. Due to the random distribution of partial discharges over many voltage half-waves, recording extends over several minutes. Parallel to each PD pulse, both the instantaneous value of the rated voltage *U_R_* and the instantaneous value of the rated current *I_R_* are recorded.

After completion of this series of measurements, the entire procedure is repeated, without the rated current being induced. The procedure described is carried out in full for each device under test (DUT) sensor. The amplitude of the measured PD pulses is used to evaluate sensitivity. Due to the sometimes strongly oscillating pulse characteristics, the peak-to-peak voltage is used as a measure of amplitude. This measure has the advantage of avoiding distortion by low-frequency signal components (e.g., 50 Hz components). For the sensitivity evaluation, only amplitude ratios are considered. Specifically, for each pulse, the DUT’s amplitude is related to the REF’s amplitude. Sensitivity is expressed as a percentage, with 100% representing the highest sensitivity of these measurements.

**Figure 11:**
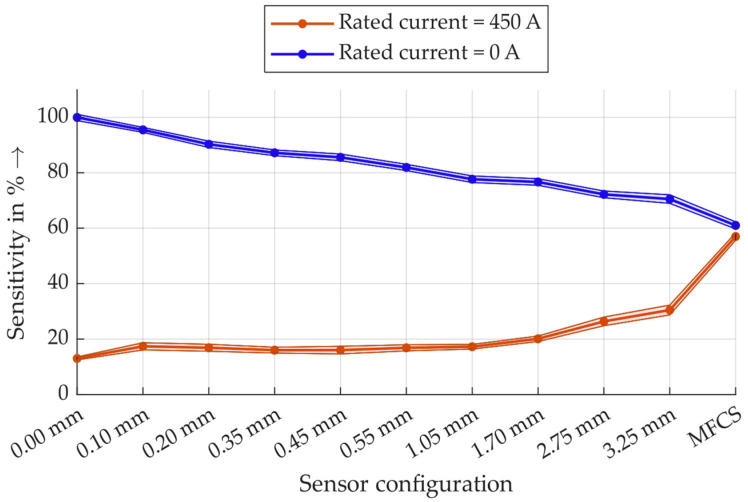
Results of the PD measurement sensitivity of all inductive sensor combinations with and without the rated current.

All measurement data summarised in an overview graph are shown in Figure 11. All sensitivity values are normalised to the largest amplitude, which is defined as 100%. Student’s t-distribution is used for statistical analysis, and the resulting 99% confidence interval limits are shown as colour-coded areas.

The measurement results show that under the rated current load, the MFCS has almost twice the sensitivity of the HFCT with the largest air gap. Without the rated current load, there is a noticeable trend that sensitivity decreases with an increasing air gap, and the MFCS has the lowest sensitivity at approximately 60%. It should be particularly noted here that the sensitivity of the MFCS under rated current is only a few percent lower than without rated current, but the difference is statistically significant. This indicates that the MFCS in this configuration is already close to the saturation range on the hysteresis curve.

**Figure 12:**
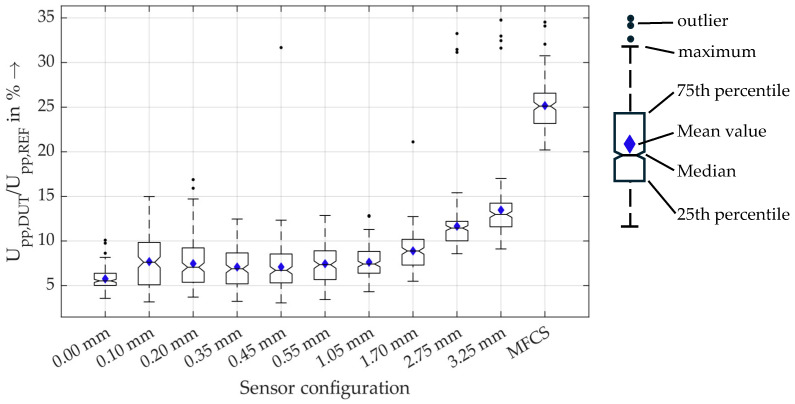
Boxplots of the relative amplitudes of the sensors under rated current load of 450 A. The boxplots show the median, the mean value and the 25th and 75th percentiles, meaning that 50% of all values are inside the box. The maximum and minimum values are marked with the dashed lines that are of a maximum length of 1.5-times the interquartile range. With a normally distributed dataset, this results in 99.3% coverage. All other values are marked as outliers.

For a closer look into the results, in Figure 12, the measurement data for all sensors are shown for the measurements with rated current of 450 A.

These results show that there is a statistically significant difference in the sensitivity of the MFCS and the HFCT with a 3.25 mm air gap (neglecting the outliers). Therefore, the MFCS concept is very beneficial for PD detection under rated current load.

**Figure 13:**
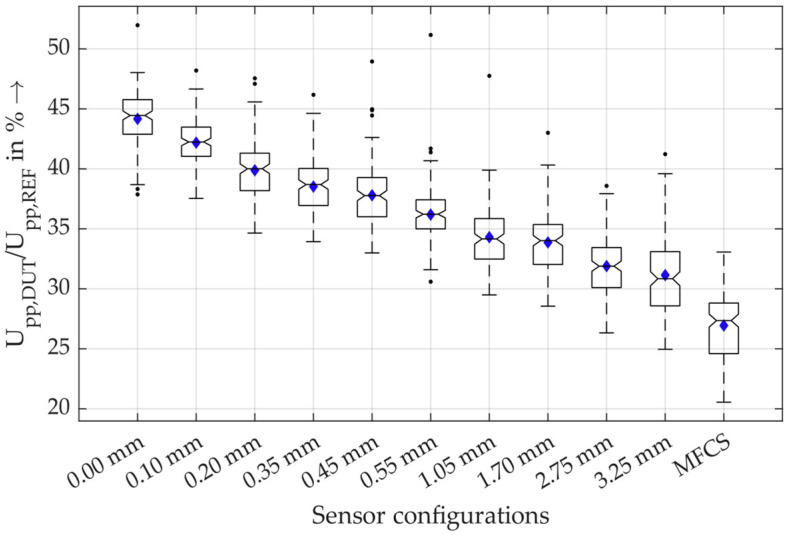
Boxplots of the relative amplitudes of the sensors without rated current load.

Furthermore, Figure 13 shows the sensitivity of the sensors without rated current load.

In contrast to the significant increase in sensitivity using MFCS at rated current load, the reduction in sensitivity without rated current load is not significant. It can, therefore, be concluded that, even under low-current conditions, the MFCS is, on average, not statistically significantly less sensitive than an HFCT. **The main feature of the MFCS is the stability of the PD detection that is independent of the load current up to 450 A. This is especially relevant for monitoring purposes and for trend analysis of PD activity.**

### 3.2. Saturation Behaviour of the MFCS in Comparison to HFCTs

The previous section showed that the MFCS loses only a small amount of sensitivity at rated current load and is insensitive to saturation effects. In the following, the specific saturation behaviour of the sensor configurations is determined as directly as possible, rather than indirectly via the sensitivity of the PD measurement. The aim is to determine a saturation current for the sensors and, thus, quantify the saturation behaviour. There are two established ways of analysing saturation properties in the literature. On the one hand, the so-called knee point voltage is used to measure transducers, while, on the other hand, total harmonic distortion (*THD*) is used to measure saturation. The knee point voltage is defined for measuring transducers in accordance with DIN EN 61869-2 [35], and THD for saturation evaluation is largely influenced by scientific publications [9,36].

**Figure 14:**
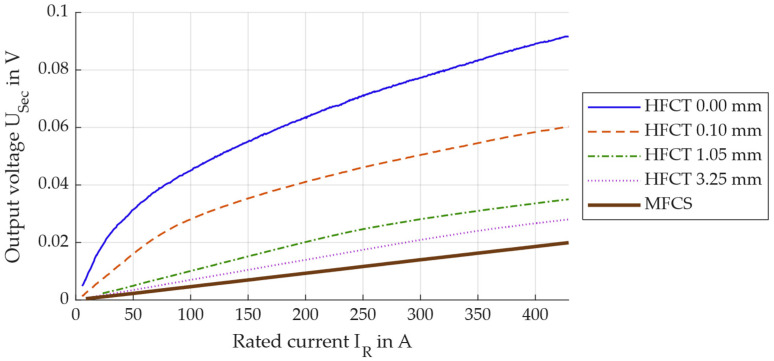
Output voltage on the secondary side of the PD sensors in dependence on the rated load current of the MV cable.

Within these measurement series, the laboratory setup remains identical to setup A used above. However, PD generation and measurement are irrelevant here, as the voltage induced on the secondary side is to be analysed as a function of the primary current. To do this, the sensor is attached to the DUT position for each sensor configuration, and the rated current *I_R_* is increased from 2 A to 450 A within 60 s. The output voltage of the HFCT and the MFCS is recorded simultaneously with the current measurement. Both the *I_R_* and the output voltage are divided into time windows, each 100 ms wide, so that five full periods are always represented and form the basis of the analysis. The acquired data are then used to calculate the effective values of the rated current and the secondary output voltage of the PD sensors for each time window. For reasons of clarity, this is only shown for selected sensor configurations in Figure 14.

The measurement data show that the secondary voltage does not rise above 100 mV as an effective value as long as less than 450 A flows on the primary side. The nonlinear behaviour for high currents is particularly evident with the HFCT 0.00 mm. Nevertheless, it is not possible to determine the knee point voltage in relation to saturation here. According to the standard, a sinusoidal voltage must be applied to the secondary terminals of the current transformer, and the resulting excitation current must be measured. The knee point is defined as a 10% increase in secondary voltage, resulting in a 50% increase in excitation current. With the sensors analysed here, this criterion is not met at any time, so that no knee point can be determined in accordance with the standard.

**Figure 15:**
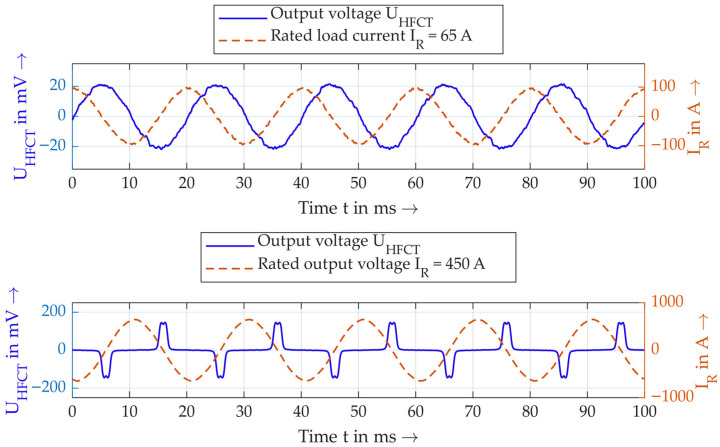
Example of distortion of the sinusoidal secondary voltage at different primary currents.

The alternative option for quantifying saturation is the method of determining the *THD*, or the distortion of the sinusoidal secondary voltage that occurs at saturation. Figure 15 shows examples of the secondary voltage and primary current for an HFCT, for two different currents.

From a qualitative point of view, it is clear that the secondary voltage becomes severely distorted at high primary currents. This is directly related to saturation, as the voltage peaks occur precisely at the zero crossings of the current, i.e., when the core is not saturated. At higher currents, the core becomes saturated and the output voltage becomes zero, as there is no change in the magnetic flux in the core over time.

The *THD* calculation is suitable for quantifying this distortion. This involves relating the sum of the amplitudes of the second to 25th harmonics to the amplitude of the fundamental according to Formula (7):

(7) $$THD=\frac{\sum_{n=2}^{25}{\left|U_{n}\right|}^{2}}{{\left|U_{1}\right|}^{2}}$$

**Figure 16:**
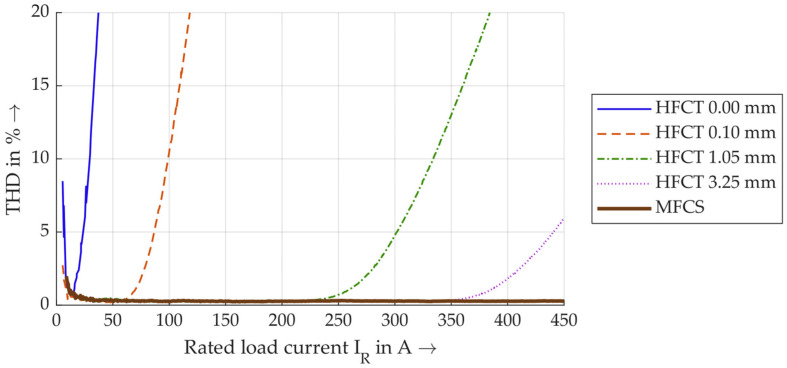
Calculated *THD* values in dependence of the load current.

If the *THD* is now determined for each 100 ms time window, the *THD* values for the same selected sensors are shown in Figure 16.

The *THD* values are given in percent and are shown here up to 20%. A larger air gap generally results in lower *THD* values. Notably, the *THD* of the MFCS remains consistent below 1% up to a maximum current of 450 A, unlike the HFCT, even with the largest air gap. It remains unclear which *THD* is used as the limit value for saturation. In [9], 1% is defined as the threshold. However, the measurements here already show higher *THD* values at very low currents below 20 A, which are not taken into account since this is due to power generation. In this work, *THDs* of 1% and 5% are used as limit values and for discussion. Table 3 lists all currents for all sensor configurations in which these thresholds are exceeded.

For most HFCT configurations, a *THD* of 5% is achieved at approx. 25–50% higher currents than a *THD* of 1%. There is also a clear trend that HFCTs with higher air gaps reach saturation later. It is particularly noteworthy that the MFCS does not exceed a *THD* of 1% at any current, whereas the HFCT 3.25 mm exceeds a *THD* of 5% at 440 A. **Overall, the measurement data show that, for rated currents of up to 450 A, the MFCS is ultimately the only sensor that does not reach saturation. This contributes to the MFCS’s property of being independent of the load current (up to 450 A), making it a reliable tool for PD trend analysis.**

### 3.3. Direction-Selective PD Detection

With the MFCS, a stable sensor is introduced that enables constant PD detection independently of the load current. The sensor is especially suitable for monitoring and trend analysis of PD. To further enhance the PD monitoring, a method for direction-selective PD detection is introduced in this study. To analyse the method, measurements are carried out using setup B, explained in Section 2.3.2. In each measurement series, the following PDs are generated one after the other, always with only one PD source at a time:

- PD at high voltage or ground potential in the switchgear, using a PD arrangement.
- PD at the beginning of the MV cable.
- PD after 10 m, 58 m or 69 m of MV cable.

High voltage is applied for each PD type, and a total of 500 PD pulses are recorded, with the same PD events being recorded by the direction detector and polarity detector. With these measured impulses, the two algorithms for direction detection (based on the Hilbert transform and the original signal) are evaluated.

**Figure 17:**
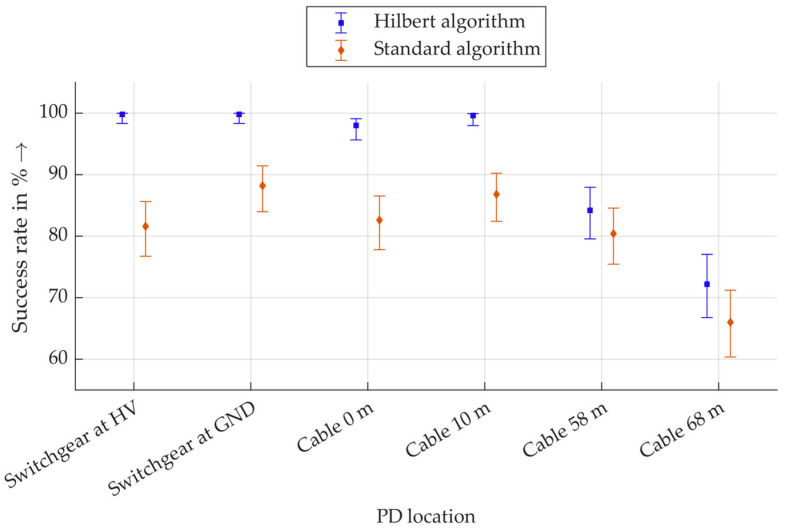
Wilson intervals of the success rates of the direction detection with the algorithms based on the Hilbert transform (Hilbert algorithm) and based on the raw signal (standard algorithm).

The result of the procedure is a directional indication relative to the direction detector. Since the position of the generated PD is known, each directional indication is assigned as either a correct or incorrect detection. Correct directional detection is defined as a success. The result is that a certain number of the 500 total PD pulses are correctly detected and evaluated as successes. From this, a relative success rate is calculated as a percentage, and a Wilson interval is calculated. The Wilson interval is a confidence interval for binary events and is particularly suitable for evaluating probabilities of success [37]. Figure 17 shows the success rates of direction detection for the Hilbert algorithm and the standard algorithm based on the raw signal.

It should be noted that the y-axis is scaled, and no values lower than 60% are displayed, as these do not exist. For all PDs in the switchgear, almost 100% of the pulses are correctly assigned with the Hilbert algorithm. The corresponding Wilson interval for a sample size of 500 values extends to approx. 98.5%. This also applies to PD at a distance of 10 m in the cable. PDs at 58 m are assigned slightly less reliably, although still over 79% of pulses are correctly located with the Hilbert algorithm. For PD at a distance of 68 m, over 66% of the pulses are still correctly assigned. This shows a trend of decreasing success rates with increasing distance. This is plausible, as PD pulses are generally more attenuated and distorted over greater distances, making correct assignments via polarity more difficult.

The directional evaluation based on the raw signals with the standard algorithm shows the same trend: the most distant PDs are less reliably assigned. The assignment of PD within the switchgear and up to a maximum distance of 10 m in the cable is correct in 80% to 90% of cases. This shows that evaluation using Hilbert transformation is more robust than evaluation based on raw data. It should be noted that the evaluation for both algorithms is performed with the same PD pulses, so that a direct comparison is permissible here. **In summary, this method provides a promising approach for determining the direction of the PD source.**

## 4. Final Discussion and Conclusions

This study introduces a novel sensor concept called MFCS, which contributes to a method for direction-selective PD detection. This concept is based on the same measurement principles as other inductive sensors, such as HFCTs. The main difference lies in the sensor’s unconventional half-ring core geometry. Measurements show that the MFCS is more sensitive than split-core HFCTs with air gaps up to 3.25 mm. Measurements analysing the saturation characteristics show that the MFCS does not saturate for currents up to 450 A. This makes this specific MFCS suitable for the mounting position around the whole MV cable, since it is not saturated from the magnetic field of the rated current. This enables stable PD monitoring independent of the rated current, resulting in constant output voltages for constant PD, unlike the analysed HFCT sensors. This is particularly important for PD trend analysis.

**Figure 18:**
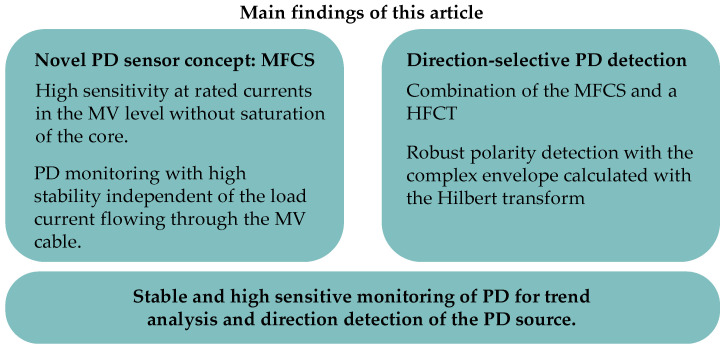
Main findings of this study.

Furthermore, the MFCS provides the basis for the direction-selective PD detection. A method is introduced and validated, where two sensors are used in combination. One is the MFCS around the entire cable and the other is a split-core HFCT around the ground wire of the cable. With the HFCT, the polarity of the PD impulses is determined, and with the MFCS, the direction is determined. This is ultimately achieved with a polarity comparison of both sensors. The results show that detecting the polarity of individual impulses by evaluating the first half-wave of these impulses is promising. A robust method for detecting the first half-wave is introduced, based on calculating the Hilbert transform and the complex envelope of the signal. The results demonstrate that the novel sensor concept is suitable for PD detection and that the novel direction detection method is promising. The main findings of this study are summarised in Figure 18.

Regarding future investigations, the methods for robust PD direction detection are to be further validated, since they reach their limitations when PDs come from more than 50 m inside the cable. Future work is planned in the area of data analysis. Overall, data analysis using the Hilbert transform shows promising results but is not yet ready for use as a prototype in the field. Future research will investigate the extent to which AI-based data analysis can improve polarity detection for highly oscillating pulses. To this end, comprehensive field tests are planned in collaboration with grid operators.

## Figures and Tables

**Table 1:** Used sensor configurations.

Sensor Type	Air Gap per Side	Total Air Gap Length
HFCT	0.00 mm	0.00 mm
HFCT	0.10 mm	0.20 mm
HFCT	0.20 mm	0.40 mm
HFCT	0.35 mm	0.70 mm
HFCT	0.45 mm	0.90 mm
HFCT	0.55 mm	1.10 mm
HFCT	1.05 mm	2.10 mm
HFCT	1.70 mm	3.40 mm
HFCT	2.75 mm	5.50 mm
HFCT	3.25 mm	6.50 mm
MFCS	-	-

**Table 2:** Correlation between the polarity of the measured impulses with fault location.

Polarity of the PD	PD Origin Location	Output Voltage of the Polarity Detector (HFCT)	Output Voltage of the Direction Detector (MFCS)
Positive	Switchgear	Positive	Positive
Positive	Cable	Positive	Negative
Negative	Switchgear	Negative	Negative
Negative	Cable	Negative	Positive

**Table 3:** Saturation currents for the used sensor configurations.

Sensor Type	Air Gap	Current for *THD* = 1%	Current for *THD* = 5%
HFCT	0.00 mm	16 A	23 A
HFCT	0.10 mm	70 A	88 A
HFCT	0.20 mm	106 A	131 A
HFCT	0.35 mm	135 A	163 A
HFCT	0.45 mm	150 A	180 A
HFCT	0.55 mm	184 A	219 A
HFCT	1.05 mm	259 A	303 A
HFCT	1.70 mm	305 A	352 A
HFCT	2.75 mm	366 A	421 A
HFCT	3.25 mm	384 A	440 A
MFCS	-	<450 A	<450 A

## Data Availability

The main data are contained within the article. Additional data are available on request from the authors.

## Abbreviations

The following abbreviations are used in this manuscript:

AC	Alternating current
DUT	Device under test
GND	Ground
HFCT	High-Frequency Current Transformer
HV	High voltage
IEC	International Electrotechnical Commission
LV	Low voltage
MFCS	Magnetic Flux Concentrator Sensor
Mn-Zn	Manganese–zinc
MV	Medium voltage
Ni-Zn	Nickel–zinc
PD	Partial discharge
PMMA	polymethyl methacrylate
PRPD	Phase-resolved partial discharge
PV	Photovoltaics
REF	Reference
TDR	Time domain reflectometry
*THD*	Total harmonic distortion
ToA	Time of arrival
